# Fungal and bacterial gut microbiota differ between *Clostridioides difficile* colonization and infection

**DOI:** 10.20517/mrr.2023.52

**Published:** 2023-12-06

**Authors:** Jannie G.E. Henderickx, Monique J.T. Crobach, Elisabeth M. Terveer, Wiep Klaas Smits, Ed J. Kuijper, Romy D. Zwittink

**Affiliations:** ^1^Center for Microbiome Analyses and Therapeutics, Department of Medical Microbiology, Leiden University Medical Center, Leiden 2333 ZA, the Netherlands.; ^2^Department of Medical Microbiology and Leiden University Center of Infectious Diseases (LU-CID), Leiden University Medical Center, Leiden 2333 ZA, the Netherlands.; ^3^Netherlands Donor Feces Bank, Department of Medical Microbiology, Leiden University Medical Center, Leiden 2333 ZA, the Netherlands.

**Keywords:** Mycobiota, fungi, gut microbiota, *Clostridioides difficile*, CDI

## Abstract

**Aim:** The bacterial microbiota is well-recognized for its role in *Clostridioides difficile* colonization and infection, while fungi and yeasts remain understudied. The aim of this study was to analyze the predictive value of the mycobiota and its interactions with the bacterial microbiota in light of *C. difficile* colonization and infection.

**Methods:** The mycobiota was profiled by ITS2 sequencing of fecal DNA from *C. difficile* infection (CDI) patients (*n* = 29), asymptomatically *C. difficile* colonization (CDC) patients (*n* = 38), and hospitalized controls with *C. difficile* negative stool culture (controls; *n* = 38). Previously published 16S rRNA gene sequencing data of the same cohort were used additionally for machine learning and fungal-bacterial network analysis.

**Results:** CDI patients were characterized by a significantly higher abundance of *Candida* spp. (MD 0.270 ± 0.089, *P* = 0.002) and *Candida albicans* (MD 0.165 ± 0.082, *P* = 0.023) compared to controls. Additionally, they were deprived of *Aspergillus* spp. (MD -0.067 ± 0.026, *P* = 0.000) and *Penicillium* spp. (MD -0.118 ± 0.043, *P* = 0.000) compared to CDC patients. Network analysis revealed a positive association between several fungi and bacteria in CDI and CDC, although the analysis did not reveal a direct association between *Clostridioides* spp. and fungi. Furthermore, the microbiota machine learning model outperformed the models based on the mycobiota and the joint microbiota-mycobiota model. The microbiota classifier successfully distinguished CDI from CDC [Area Under the Receiver Operating Characteristic (AUROC) = 0.884] and CDI from controls (AUROC = 0.905). *Blautia* and *Bifidobacterium* were marker genera associated with CDC patients and controls.

**Conclusion:** The gut mycobiota differs between CDI, CDC, and controls and may affect *Clostridioides* spp. through indirect interactions. The mycobiota data alone could not successfully discriminate CDC from controls or CDI patients and did not have additional predictive value to the bacterial microbiota data. The identification of bacterial marker genera associated with CDC and controls warrants further investigation.

## INTRODUCTION

*Clostridioides difficile* is an anaerobic, Gram-positive, spore-forming bacterium and the main causative organism of nosocomial diarrhea. Upon secretion of toxin A and/or B, and in some strains binary toxin, colonic inflammation is induced, giving rise to clinical manifestations that range from mild diarrhea with abdominal cramping to life-threatening pseudomembranous colitis, toxic megacolon, and death^[1]^.

The disruption of the microbiome due to antibiotic use is a major risk factor for *C. difficile* infection (CDI), as is an age of 65 years and above^[1]^. In CDI patients, the bacterial microbiome displayed low diversity and decreased abundance of *Bacteroides*, *Prevotella*, and *Bifidobacterium* species, while exhibiting an elevated abundance of *Clostridioides* and *Lactobacillus* species^[2,3]^. It remains unknown whether these microbial changes are associated with risk factors leading to CDI, such as antibiotic use, or due to the presence of *C. difficile* in the gut^[4]^. Fecal microbiota transplantation (FMT) is a highly effective treatment for recurrent CDI (rCDI) that re-establishes a healthy gut microbial community, inhibits the growth of *C. difficile*, and prevents recurrence^[5-10]^. Microbiome-based therapies for rCDI are currently focused on restoration of the gut microbiota with bacterial products and do not include fungi, although the yeast *Saccharomyces boulardii* may be a promising prophylactic^[11,12]^.

Although *C. difficile* can lead to (recurrent) infections, the organism can also be part of the human gut microbiota without causing symptoms. Such asymptomatic colonization is estimated to occur between 0% and 15% of the healthy adult population^[13]^. Carriage of *C. difficile* is potentially a risk factor for transmission to a susceptible population, and because of the bacterium’s opportunistic nature, it may progress to infection under circumstances in which the microbiome is disturbed^[14-18]^, though carriage does not contribute to transmission in a setting with low prevalence of hypervirulent PCR ribotype 027^[19]^.

Aside from bacteria, fungi are an integral part of the microbiome. Recent research has emphasized the role of fungi in human health^[20]^. In CDI specifically, the gut mycobiota has been shown to deviate from *C. difficile* carriers and from patients with diarrhea that is not attributable to CDI^[21-24]^. In fact, fungal OTUs combined with host immune markers could successfully distinguish CDI patients from carriers with machine learning^[22]^. The mycobiota of CDI patients are characterized by lower biodiversity, an increased ratio of Ascomycota to Basidiomycota, and decreased abundance of *Saccharomyces*, *Cladosporium*, and *Aspergillus*^[21-23,25]^. Additionally, the effectiveness of FMT for the treatment of CDI has been positively associated with the genera *Saccharomyces* and *Aspergillus* and negatively associated with the dominance of *Candida albicans*^[26]^.

Importantly, *C. difficile* was shown to withstand otherwise toxic aerobic conditions when cultured in the presence of *C. albicans*^[27]^. Further supporting the importance of fungal-bacterial interactions, it was shown that *C. difficile*-directed antibiotics, including metronidazole and vancomycin, lead to the outgrowth and emergence of potentially pathogenic fungi^[25]^. As such, an intriguing triad of antibiotics, bacteria, and fungi exists in *C. difficile* colonization and infection^[28]^.

Despite the evidence suggesting fungal-bacterial interactions in CDI, the predictive value of the gut mycobiota alone is scarcely investigated and unexplored in combination with the bacterial microbiota. Studying the mycobiota composition, its predictive value for *C. difficile* asymptomatic colonization and (recurrent) infection, and interactions with the bacterial microbiota could contribute to the overall understanding of the changes associated with the presence of *C. difficile* in the intestinal tract. Such knowledge may help understand the value of fungi in diagnosing and treating CDI and may be of interest for future therapeutic microbiome intervention strategies. In the present study, we evaluated whether mycobiota data alone or in combination with bacterial composition data allows discrimination of *C. difficile* carriers from hospitalized, non-colonized controls or CDI patients.

## METHODS

### Subjects and sample collection

Samples were derived from a multicenter prospective, hospital-based, case-control study performed in the Netherlands, as described earlier^[29]^. The aim of that study was to assess the prevalence of *C. difficile* colonization at hospital admission and the risk of subsequent infection or onward transmission^[19]^. A subset of samples from this study from two of the participating hospitals [Leiden University Medical Centre (LUMC), a tertiary academic hospital; Amphia Hospital, a large general hospital] was included in our analysis.

Patients were part of one of three study groups: patients with CDI, asymptomatic *C. difficile* colonization (CDC), and patients without *C. difficile* colonization (controls). The majority of the patients from each group had received antibiotic treatment up to three months prior to sample collection^[29]^ [Supplementary Table 1]. Feces was collected within 72 h of hospital admission and cultured for *C. difficile*. Suspicious colonies were tested to confirm the presence of *C. difficile* and free toxins as previously described^[19,29]^. Patients with a positive *C. difficile* culture but no diagnosis of CDI within the first 72 h of admission were considered *C. difficile* colonized. Patients with a negative stool culture at hospital admission were included as controls. Samples of the CDI group were derived from patients hospitalized at the LUMC and diagnosed with CDI during the study period. CDI diagnosis was based on strict clinical criteria in combination with laboratory CDI testing (including detecting free *C. difficile* toxins and culturing). *C. difficile* isolates from CDI and CDC were additionally ribotyped with capillary PCR^[30]^. The identified ribotypes among CDI and CDC patients are described in Supplementary Figure 1.

The institutional review board of LUMC and the directing board of the Amphia Hospital had no objection to the performance of the study. A waiver for informed consent for feces collection of CDI patients was obtained. Feces of CDC and controls were collected under verbal consent; written informed consent was obtained for the collection of additional data.

### Microbiota analysis

Microbiological and microbiota analyses have been performed and processed previously^[29]^. In this study, these data were supplemented with Internal Transcribed Spacer (ITS) 2 sequencing data. ITS2 sequencing was performed on available samples (Total: *n* = 105; CDI: *n* = 29; CDC: *n* = 38; controls: *n* = 38), and corresponding samples were selected from the available and processed 16S rRNA sequencing data.

### Mycobiota analysis

#### DNA extraction, library preparation and sequencing

Based on sample availability, mycobiota profiling was performed on 105 of the 125 fecal samples, on which 16S rRNA gene sequencing was performed^[29]^. DNA extraction, quality control, library preparation, and ITS2 sequencing were performed according to the standard operating procedures of BaseClear B.V. (the Netherlands). DNA was extracted by a combination of chemical and mechanical lysis, in which two empty tubes were included as negative controls and two cellular microbial standards as positive controls (D6300 ZymoBIOMICS Microbial Community standard, Zymo Research, USA). The microbial standard contains ten microbial strains, of which two are fungi: *Saccharomyces cerevisiae* and *Cryptococcus neoformans*. After DNA extraction, concentrations were measured with Quant-iT dsDNA Assay kits [high sensitivity (Q33120) and broad range (Q33130), Invitrogen, USA], and DNA integrity was checked on an agarose gel. Subsequently, the ITS2 region was PCR-amplified with primers ITS3 (Forward: 5’-GCATCGATGAAGAACGCAGC-3’) and ITS4 (Reverse: 5’-TCCTCCGCTTATTGATATGC-3’), resulting in a ~490 bp amplicon complemented with standard Illumina adapters. Unique Index Primers were attached to amplicons in each sample with a second PCR cycle. PCR products were purified using Agencourt AMPure XP (Becker Coulter, USA) and DNA concentration was measured by fluorometric analysis (Quant-it dsDNA Assay Broad Range kit, Invitrogen, USA). Next, PCR amplicons were equimolarly pooled. Libraries were prepared with Nextera XT Index Kit v2 (FC-131-2004, Illumina, USA), checked on concentration with Quant-iT dsDNA Assay High Sensitivity kit, and on size with Agilent DNA 1000 (Agilent, USA). After quality control of the library, ITS2 amplicon sequencing was performed on the MiSeq platform (Illumina, USA) with 300 bp paired-end reads. To control for the sequencing process, a microbial DNA standard was included (D6305 ZymoBIOMICS Microbial Community DNA standard, Zymo Research, USA).

#### Bioinformatic analyses: taxonomic assignment

Raw reads were processed according to the Q2-ITSxpress workflow^[31]^. Raw reads without barcodes and primers were imported in Qiime2 (version 2022-2)^[32]^. Subsequently, the conserved regions around the ITS gene were trimmed with ITSxpress, which has been shown to improve the accuracy of taxonomic classification^[33]^. The sequence variants were then identified in the unmerged, trimmed sequences with DADA2^[34]^. Next, the Qiime classifier was trained using the UNITE database (version 8.3, all eukaryotes)^[35]^. Fungal ITS classifiers were trained on the UNITE database on full reference sequences. Subsequently, sequence variants were classified with the trained classifier.

#### Bioinformatic analyses: pre-processing data

Data were imported in R (version 4.1.2)^[36]^ with the *Qiime2R* package (version 0.99.6)^[37]^. In all samples, 959 taxa were identified, with 1,721,901 reads in total. The mean number of reads was 15,513 and ranged between a minimum of 255 and a maximum of 59,126. The sum of reads of three samples were outliers (≤ 380 reads). These three samples were retained since one of those samples was a negative control (NC1) and two samples were derived from CDI patients who received antibiotics in the past three months. Pre-processing included filtering of 491 non-fungal taxa belonging to the kingdoms Viridiplantae and Metazoa. The fungal kingdom and unassigned reads were retained and resulted in a mean number of 10,669 reads per sample, with a minimum of 21 and a maximum of 54,775.

#### Bioinformatic analyses: data quality control

Positive controls were included during DNA extraction (PCE) and sequencing (PCS), as well as negative controls for DNA extraction (NC) [Supplementary Figure 2]. The total number of reads from the filtered ASV table was visualized with *ggplot2* (version 3.3.6)^[38]^. The mean total reads of positive (22.915 ± 2.686) and negative (748 ± 1.029) DNA extraction controls were not significantly different (*P* = 0.33, Wilcoxon Rank Sum Test, Supplementary Figure 2A). One of the negative controls, NC2, contained *Malassezia globosa*, which is often identified on human skin^[39]^, as well as *Cyberlindnera jadinii* (teleomorph of *Candida utilis*) that has been isolated from distinct environments^[40]^ [Supplementary Figure 2B].

Additionally, taxonomic compositions of positive controls were compared to the theoretical mock community. The two genera of the mock community - *Cryptococcus* and *Saccharomyces* - are present in a 1:1 ratio in the mock community, indicating a mean overrepresentation (1.48-fold) of *Cryptococcus* spp. and a mean underrepresentation (0.52-fold) of *Saccharomyces* spp. in the DNA and sequencing positive controls [Supplementary Figure 2C]. Correlation coefficients between the positive controls of DNA extraction (ρ = 1.00, *P* ≤ 0.001) and between positive controls of sequencing (ρ = 0.99, *P* ≤ 0.001) indicate high reproducibility between batches.

#### Bioinformatic analyses: downstream analyses

Pre-processed reads were used for downstream analyses in R (version 4.1.2)^[36]^.

The number of reads and relative abundances from the ASV table were used to generate composition plots with *microbiome* (version 1.16.0)^[41]^ and *ggplot2* (version 3.3.6)^[38]^.

Shannon diversity was computed at the ASV level with *microbiome* after reads had been rarified to the minimum sum of reads per sample with *phyloseq* (version 1.38.0)^[42]^. Overall significant differences were computed with a Kruskal-Wallis test from *stats* (version 4.1.2)^[36]^. *P-*values below 0.05 were considered statistically significant.

Differential abundant features were identified with *ALDEx2* (version 1.26.0)^[43]^ using centered log-ratio transformed values and default parameters, comparing CDI with controls and CDI with CDC at genus and species levels. The Wilcoxon Rank Sum Test of *ALDEx2* was used for significance testing with Benjamini-Hochberg corrected *P*-values. *P-*values below 0.05 were considered statistically significant. Relative abundance of the differential abundant features was visualized for the three study groups and means were compared with the Wilcoxon Rank Sum Test of *ggpubr* (version 0.4.0)^[44]^.

Machine learning was performed at the OTU and ASV level with the SIAMCAT package (version 2.1.3)^[45]^ on 16S rRNA and ITS2 gene amplicon sequencing separately, as well as on the joint data. The CDI group was compared to the CDC group and to controls who received antibiotic treatment. Unsupervised feature filtering was performed with default parameters. Data was normalized with log.unit normalization and normalization parameters log.n0 = 1e-06, n.p = 2 and norm.margin = 1. A twice-repeated 5-fold cross-validation scheme was generated and the model was trained using the lasso method.

Fungal-bacterial network analyses were performed with SParse InversE Covariance Estimation for Ecological Association Inference, *SpiecEasi* (version 1.1.2)^[46]^ combining 16S rRNA and ITS2 amplicon sequencing data with *multi.spiec.easi*. Prior to the network analyses, taxa were aggregated to genus level and pruned at 100 reads using *microbiome* and *phyloseq*. Network analyses for each study group were performed using meinshausen-Buhlmann's neighborhood selection with lambda.min.ratio = 0.01, nlambda = 20 and rep.num = 99. Networks were visualized with *ggraph* (version 2.0.5)^[47]^.

## RESULTS

### *C. difficile*-infected patients are enriched with *Candida* spp.

**Figure 1:**
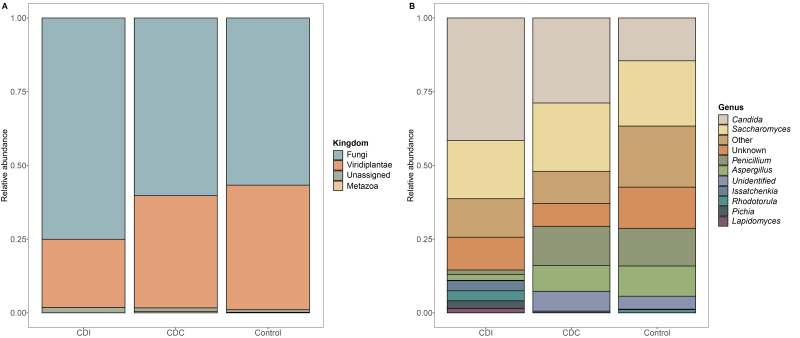
Taxonomic profiles of CDI patients, asymptomatic CDC patients, and controls (Control). (A) Eukaryotic taxonomic profiles at kingdom level are shown for the three study groups; (B) Fungal taxonomic profiles on genus level are shown for the ten most abundant genera. Taxa not belonging to the ten most abundant genera were summarized into “Other”. CDC: *C. difficile* colonization; CDI: *C. difficile* infection.

To assess differences in the mycobiota of CDI, CDC, and controls, taxonomic profiles of the mycobiota using ITS2 sequencing were generated [Figure 1]. This revealed the presence of eukaryotic kingdoms, including Fungi, Viridaeplantae, and Metazoa. [Figure 1A]. For further analyses, fungal and unassigned kingdom reads were retained and comprised 468 out of 959 taxa and 68.8% of total reads. The relative abundance of the fungal kingdom was 0.75, 0.60, and 0.57 in CDI, CDC, and controls, respectively (± 0.32, 0.39, and 0.39 S.D.).

**Figure 2:**
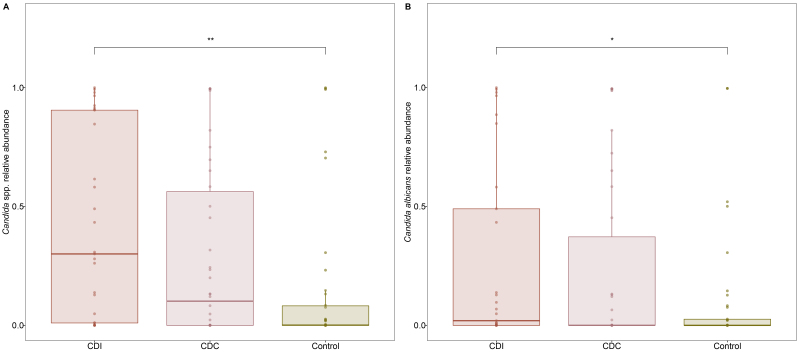
Relative abundance of *Candida* spp. (A) and *Candida albicans* (B) in CDI patients, asymptomatic CDC patients, and controls (Control). Individual data points are displayed as circles. ^**^P ≤ 0.01; ^*^P ≤ 0.05 (Wilcoxon Rank Sum Test). CDC: *C. difficile* colonization; CDI: *C. difficile* infection.

The most abundant genus in CDI patients was *Candida* spp. [Figure 1B], with a significant mean difference (MD) compared to controls (Figure 2A, MD 0.270 ± 0.089, *P* = 0.002, Wilcoxon Rank Sum Test). More specifically, the mean relative abundance of *C. albicans* was higher in CDI patients compared to controls (Figure 2B, MD 0.165 ± 0.082, *P* = 0.023). This was accompanied by a decrease in *Saccharomyces* spp., with a significantly higher *Candida* to *Saccharomyces* ratio in CDI patients compared to controls (Supplementary Figure 3, *P* = 0.024, Wilcoxon Rank Sum Test). The *Saccharomyces* genus was underrepresented in the mock community, which may overestimate the shift in the ratio. Moreover, the mean relative abundance of the genera *Aspergillus* and *Penicillium* was significantly lower in CDI patients compared to CDC patients and controls [Supplementary Figure 4].

Differential abundance testing confirmed the significant increases in CDI patients of *Candida* spp. (*P_unadj_* = 0.003) and *C. albicans* (*P_unadj_* = 0.027) compared to the controls, as well as the significant deprivation of *Aspergillus* (*P_adj_* = 0.044) and *Penicillium* species (*P_unadj_* = 0.002) compared to CDC patients. No differential abundant features were identified between CDC patients and controls.

Besides differences in the mycobiota composition, the median Shannon diversity increased from CDI patients to CDC patients to controls, though differences between groups were not significant (Supplementary Figure 5, *P* = 0.167, Kruskal-Wallis test).

Together, these results indicate that CDI patients were most deviant from CDC patients and controls regarding the taxonomic composition and the diversity of the mycobiota, even though the majority of the patients from each group had received antibiotic treatment up to three months prior to sample collection^[29]^.

### The microbiota can discriminate *C. difficile* infection from colonization

Machine learning was used to investigate if models of the microbiota, mycobiota, and joint data could discriminate between CDI, CDC, and controls. With regard to the controls, antibiotic-treated controls were selected to exclude bias introduced by antibiotic treatment. The performance of the models was evaluated based on the Area Under the Receiver Operating Characteristic (AUROC), indicating the probability of classifying a randomly selected patient to the correct study group.

First, the bacterial microbiota, mycobiota, and joint models were used to assess their performance in discriminating between CDI and CDC patients, as well as between CDI patients and controls [Supplementary Figure 6A-C]. Comparing CDI and CDC patients, the AUROC of the mycobiota (0.627, 95%CI: 0.491-0.764) was significantly lower compared to that of the bacterial microbiota (0.884, 95%CI: 0.808-0.960) [Supplementary Figure 6A]. Similar results were obtained when CDI patients were compared to controls (AUROC mycobiota: 0.632, 95%CI: 0.469-0.795, AUROC microbiota: 0.905, 95%CI: 0.816-0.991) [Supplementary Figure 6B]. Moreover, the joint model did not significantly differ from the bacterial microbiota models when comparing CDI to CDC patients (0.865, 95%CI: 0.782-0.948) and CDI to antibiotic-treated controls (0.897, 95%CI: 0.810-0.984) [Supplementary Figure 6A and B]. Overall, these results indicate that the bacterial fraction of the microbiota is most successful in discriminating between CDI and CDC patients and between CDI patients and controls.

**Figure 3:**
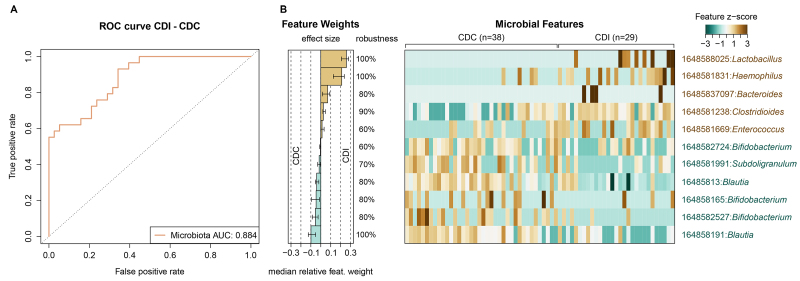
Signature of gut bacterial OTUs associated with CDI patients and asymptomatic CDC patients. (A) Cross-validation accuracy of the microbiota classifier is shown in the ROC curve with the AUC; (B) Median relative feature weights of the selected gut bacterial OTUs show the contribution of each marker OTU to the classification. Robustness of the selected features indicates the fraction of models containing the specific feature. The normalized values of the selected features across CDI and CDC are shown in the heatmap. AUC: Area Under the Curve; CDC: *C. difficile* colonization; CDI: *C. difficile* infection; ROC: Receiver Operating Characteristic.

The two microbiota models with an AUROC above 0.800 were selected to further assess the bacterial OTUs that were collectively characteristic for CDI and CDC patients [Figure 3] and for CDI patients and controls [Supplementary Figure 7]. In both cases, *Clostridioides* was identified as a distinct taxon in CDI patients. The nucleotide sequence of this *Clostridioides* OTU (OTU 1648581238) demonstrated a 100% sequence identity with *Clostridioides difficile*. Compared to CDC, CDI patients were additionally characterized by the genera *Lactobacillus*, *Haemophilus*, *Bacteroides*, and *Enterococcus* [Figure 3B]. Interestingly, CDC and controls shared OTUs from the *Bifidobacterium* and *Blautia* genera. The nucleotide sequences belonging to the identified *Bifidobacterium* OTUs resulted in 100% sequence identity with species *Bifidobacterium breve* and *Bifidobacterium longum* with subspecies *longum*, *infantis*, and *siullum*, while those belonging to the identified *Blautia* OTUs resulted in 100% sequence identity with *Blautia wexlerae*, *Blautia luti*, and *Blautia massiliensis*. In the CDC patients, *Subdoligranulum* was additionally part of the microbial signature [Figure 3B], while *Collinsella* was associated with the signature in the controls [Supplementary Figure 7B]. Together, these results demonstrate that CDC patients could not be successfully distinguished from controls based on machine learning with bacterial data. In comparison to CDI, *Bifidobacterium* and *Blautia* were bacterial marker genera for asymptomatic *C. difficile* carriage and non-carriage.

### Fungi and bacteria interact in *C. difficile* infected and colonized patients

**Figure 4:**
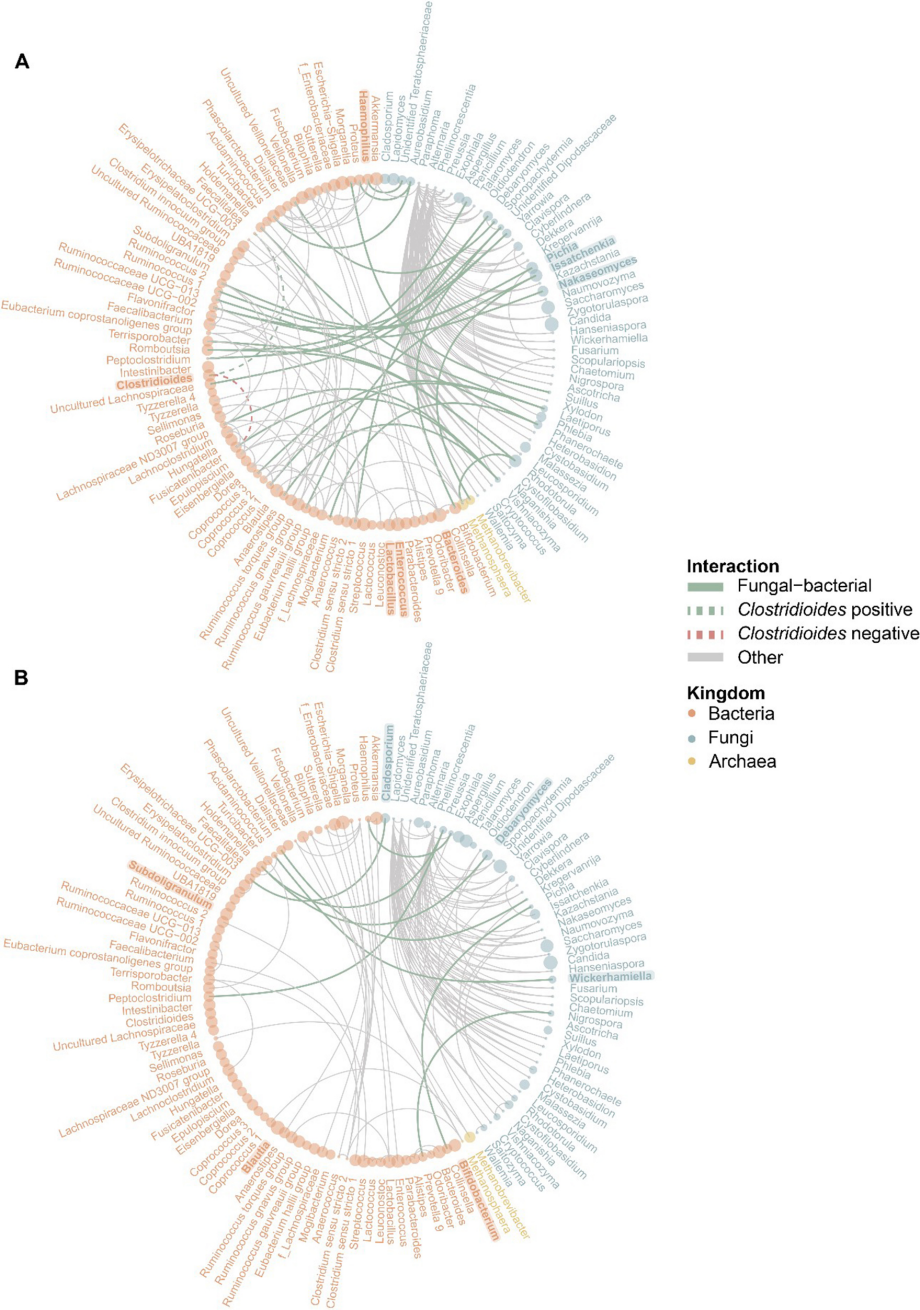
Fungal-bacterial networks in CDI patients (A) and asymptomatic CDC patients (B). Interactions between fungi and bacteria are highlighted in green, while other interactions are displayed in grey. Kingdoms are represented by color; the mean relative abundance of the genus is represented by point size. Bacterial taxa in bold are part of the microbial signature as identified by machine learning. Fungal taxa in bold are part of the three most abundant fungi in the respective study group. (A) In CDI patients, interactions with the *Clostridioides* genus are additionally displayed. CDC: *C. difficile* colonization; CDI: *C. difficile* infection.

Fungal-bacterial network analysis using SParse InversE Covariance Estimation for Ecological Association Inference (SpiecEasi) was performed for the CDI and CDC patients [Figure 4] and for the controls [Supplementary Figure 7]. The outcomes of the network analyses were used in combination with the machine learning results to infer potential associations. Interactions were considered for the three most highly abundant fungi of the group that showed interactions with bacteria, as well as for the identified bacterial marker genera [Figure 3 and Supplementary Figure 7]. In CDI and CDC patients, bacteria and fungi were only positively associated with each other.

In CDI patients, twenty-nine fungal-bacterial associations were observed [Figure 4A]. Abundant fungi positively associated with bacteria included interactions between *Pichia* and *Ruminococcus* 2; *Nakaseomyces* and *Ruminococcaceae* UCG-002; and between *Issatchenkia* and *Ruminococcus gauvreauii* group. Interestingly, no associations were observed for the *Candida* genus. From the bacterial perspective, fungal-bacterial associations were observed for the bacterial marker genera associated with CDI [Figure 3]. *Haemophilus* was positively associated with an unidentified species of the *Teratosphaeriaceae* genus. Yet, the *Clostridioides* genus did not associate with fungi, but did show a negative association with *Fusicatenibacter*. Additionally, no fungal-bacterial associations were observed for the CDI bacterial marker genera *Bacteroides*, *Bifidobacterium*, and *Lactobacillus*, although *Bacteroides* did negatively associate with *Bifidobacterium* and *Lactobacillus* [Supplementary Figure 8A].

Nine fungal-bacterial associations were observed in CDC patients [Figure 4B]. Associations between the abundant fungi and bacteria included interactions between *Debaryomyces* and *Holdemanella*; *Cladosporium* and *Erysipelotrichaceae* UCG-003; and *Wickerhamiella* and *Acidaminococcus*. Similar to CDI, *Candida* spp. did not show any associations. From the bacterial viewpoint, no interactions were observed for the CDC bacterial marker genera [Supplementary Figure 8B].

In the controls who received antibiotics, *Collinsella* and *Bifidobacterium* were few among the bacterial marker genera [Supplementary Figure 7]. *Collinsella* abundance was positively associated with *Anaerostipes*, while *Bifidobacterium* negatively associated with *Aspergillus* [Supplementary Figure 8C].

## DISCUSSION

Here, we show distinct fungal profiles in CDI compared to CDC and controls, most notably enrichment of *Candida* spp. and deprivation of *Aspergillus* spp*. and Penicillium* spp. in CDI. We also describe that mycobiota data alone could not successfully discriminate CDC from controls or CDI patients. Lastly, fungal-bacterial networks showed no association for *Candida* and no fungal-bacterial associations for *Clostridioides* in CDI and CDC. A negative bacterial-fungal association was observed between *Bifidobacterium* and *Aspergillus* in controls.

There is contradictory evidence for the role of *Candida* spp. in CDI in literature. Some studies are suggestive of a protective effect of *Candida* in CDI^[48-50]^. A lower prevalence of *Candida* colonization was observed in CDI patients^[48,49]^, and susceptibility to CDI was reduced upon *C. albicans* colonization prior to a lethal challenge with *C. difficile*^[50]^. In contrast to the protective effect of *Candida* spp., other studies report an increase in *Candida* spp. in CDI^[22,23,26]^. Specifically, *C. albicans* could be involved in the pathogenesis of CDI by allowing *C. difficile* to thrive^[27]^, reducing the healing of inflammatory lesions^[51]^, driving Th17-mediated immune responses, and disrupting the gut microbiome^[20]^. In line with the pathogenic role of *Candida* spp. in CDI, a high prevalence of *C. albicans* was reported upon infection with *C. difficile* hypervirulent PCR ribotype 027^[52,53]^. Within our cohort, differential ribotype profiles were observed in CDI and CDC patients. Interestingly, the *C. difficile* binary toxin positive ribotype 078 was more frequently found in CDI patients compared to CDC patients (24.10% *vs.* 2.63%, *P* = 0.017, Fisher’s Exact Test), but the number of patients was not sufficiently high to analyze this group separately. The differential ribotype profiles may affect the mycobiota compositions and interactions with the host, although this triad of interactions has not been assessed hitherto. Our findings corroborate the pathogenic hypothesis, showing a higher mean relative abundance of *Candida* spp. and *C. albicans* in CDI patients compared to controls, which was accompanied by an increase in the ratio between *Candida* spp. and *Saccharomyces* spp. Antibiotic treatment is commonly associated with the overgrowth of *Candida* species^[54,55]^. Within our study, 96.6% of CDI and 60.5% of CDC patients received antibiotic treatment in the last three months prior to fecal sample collection. When we compared antibiotic-treated CDI patients (*n* = 28) to antibiotic-treated controls (*n* = 17), the mean relative abundance of *Candida* spp. was still significantly higher in CDI patients (*P* = 0.021, data not shown). This suggests *Candida* spp. could be involved in the pathogenesis of CDI rather than being an effect of antibiotic treatment *per se*. Nevertheless, the contradicting findings on the role of *Candida* spp. reflect the highly variable nature between patients and might, therefore, limit the use of *Candida* spp. as a marker for CDI.

Other fungi also show differential abundance between CDI, CDC, and controls. CDI patients were deprived of *Aspergillus* and *Penicillium*. *Aspergillus* has previously been related to healthy status^[22,23,26]^ and therefore highlights its potentially beneficial role against CDI. Hypothetically, *Aspergillus* could interact with the immune system by affecting cytokine levels, as this genus was previously negatively correlated to IL-4 and TNF-α serum immune levels in a non-CDI group^[22]^. Moreover, the significant reduction of the *Candida* to *Saccharomyces* in controls might point towards a protective effect, as the fungal probiotic *S. boulardii* has been associated with a reduced risk of *C. difficile*-associated diarrhea^[11,56]^. Moreover, its supplementation during antibiotics prevented the development of CDI and reduced rCDI. Yet, evidence is insufficient for the clinical application of *S. boulardii* as a probiotic^[57]^.

Although fungi have been studied as a marker for CDI in combination with host immune factors^[23]^, this study provides novel information by combining mycobiota and microbiota data in a well-defined cohort of *C. difficile* infection patients, asymptomatic *C. difficile* colonization patients and hospitalized non-colonized controls. As opposed to CDI patients, CDC patients and controls were more alike in their bacterial and fungal microbiome. In fact, CDC patients could not be successfully distinguished from controls based on machine learning with bacterial data alone or when combined with fungal data. *Bifidobacterium* spp. and *Blautia* spp. were bacterial marker genera associated with both CDC and controls. In line with these findings, other studies have reported depletions of *Bifidobacterium* and *Blautia* in CDI patients compared to non-CDI patients^[58]^ and were associated with less severe CDI-associated outcomes^[59]^. Among the *Bifidobacterium* OTUs, *Bifidobacterium longum* subsp. *longum* has been identified as a predictor for negative *C. difficile* status^[60]^. Both *Bifidobacterium* and *Blautia* include species that are known to be involved in bile acid metabolism and short-chain fatty acid production^[61,62]^. In most of the bifidobacterial marker OTUs, bacterial bile salt hydrolase (BSH) genes have been identified. Such mechanisms could contribute to colonization resistance against *C. difficile*^[9,63-65]^.

The combination of machine learning and network analyses can infer potentially important fungal-bacterial interactions in CDI and CDC patients. Interestingly, the abundance of *Clostridioides* was not associated with fungi in CDI and CDC in these analyses, while it did identify the previously reported negative association with the Gram-positive *Fusicatenibacter* genus in CDI^[29,66]^. Moreover, the CDI-associated genus *Bacteroides* showed a negative association with *Bifidobacterium*. As *Bifidobacterium* was a bacterial marker genus associated with CDC patients and controls, increased relative abundance of *Bacteroides* could have a negative impact on beneficial bacterial-fungal interactions such as those between *Bifidobacterium* and *Aspergillus* as observed in antibiotic-treated controls. Although explorative, the fungal-bacterial network analyses described herein provide insight into differential interactions that occur during *C. difficile* colonization and infection.

FMT has proven highly effective for the treatment of rCDI, in which the restoration of the bacterial microbiota undoubtedly contributes to the successful eradication of CDI symptoms^[9]^. Emerging data suggest similar roles for the mycobiota. Increased fungal engraftment rates were observed in rCDI patients who responded to FMT compared to patients who did not respond to FMT^[26]^. More specifically, the genera *Aspergillus*, *Penicillium*, and *Saccharomyces* were significantly higher in responders post-FMT, while a dominance of *Candida* and *C. albicans* was observed in non-responders post-FMT^[26]^. Additionally, the efficacy of FMT in clearing *C. difficile* infection could be restored when *C. albicans*-colonized mice were treated with the antifungal agent fluconazole before FMT^[26]^. Within this study, CDI patients were depleted of *Aspergillus*, *Penicillium*, and *Saccharomyces* species, while the relative abundance of *Candida* spp. and *C. albicans* was increased. Although the mycobiota did not have a predictive role in distinguishing CDI, CDC, and controls, gut fungi might play a role in FMT effectiveness by interacting with the bacterial community of the gut microbiota.

It was noted in our previous work that patient groups may not be comparable with regard to solid organ transplants, previous hospitalization, immunosuppressant use, and chemotherapy, as the majority of diagnosed CDI patients were recruited at the university-affiliated hospital (LUMC), whereas CDC patients and controls were more evenly recruited at a general hospital (Amphia hospital) and the LUMC^[29]^. Several of those clinical variables affect microbiota composition^[67,68]^, yet its effect on the mycobiota remains to be elucidated. In a subgroup analysis on LUMC patients, results remained similar (data not shown). While the relative abundance of *Candida* spp. and *C. albicans* was not significantly higher in CDI compared to all controls, the mean ratio between *Candida* spp. and *Saccharomyces* spp. remained significantly higher in CDI compared to antibiotic-treated controls (*P* = 0.018). Moreover, CDI patients were, on average, depleted of *Aspergillus* spp. (*P* = 0.003) and *Penicillium* spp. (*P* = 0.015) compared to all controls and to CDC patients, respectively. The relative abundance of specific fungal genera, such as *Candida* spp., might thus be affected by the aforementioned clinical variables. In addition to that, the history of antifungal use remained unregistered, but this may have impacted the mycobiota composition significantly. Yet, fungal infections are uncommon in patients at risk for CDI in The Netherlands, except for severely immunocompromised patients and ICU patients receiving antifungal agents as part of selective gut decontamination. Moreover, 16S rRNA gene sequencing and ITS2 sequencing were performed separately. For ITS sequencing specifically, sequencing the full ITS region may provide greater taxonomic resolution compared to sequencing the ITS1 or ITS2 region alone^[69]^. However, future studies should rely on shotgun metagenomic sequencing to characterize additional kingdoms as well as genes to perform functional characterization of the microbiota and mycobiota. The characterization of additional kingdoms could be especially relevant to investigate the role of the relatively understudied Archaea and bacteriophages, which gained increasing attention with regard to FMT efficacy in CDI patients^[70]^. Lastly, the identification of bacterial markers for *C. difficile* infection patients, asymptomatic *C. difficile* colonization patients, and hospitalized non-colonized controls relied upon machine learning, which typically requires voluminous data^[71,72]^. Given the relatively low sample number per group, it would be desirable to consolidate our findings by testing the trained model on similar cohorts and to further stratify between first-onset and recurrent CDI.

In conclusion, we have shown that the gut mycobiota differs between *C. difficile* infection, asymptomatic carriage, and non-carriage. Most notably, *C. difficile* infected patients were enriched in *Candida* spp. and deprived of *Aspergillus* spp*. and Penicillium* spp. The mycobiota data alone could not successfully discriminate *C. difficile* carriers from hospitalized, non-colonized controls or CDI patients and did not have additional predictive value to the bacterial microbiota data. While not valuable as a diagnostic tool, indirect fungal-bacterial interactions may affect *Clostridioides* spp. and subsequent treatment effectiveness. The identification of bacterial marker genera associated with carriage and non-carriage warrants further investigation for diagnosing and treating CDI.

## DECLARATIONS

### Availability of data and materials

The raw ITS (mycobiota) and 16S rRNA (microbiota) gene amplicon sequencing data have been deposited at the European Nucleotide Archive (ENA) at EMBL-EBI under accession number PRJEB61263 and PRJEB30586, respectively.

### Copyright

© The Author(s) 2023.
