# Supplementary material for: Fungal and bacterial gut microbiota differ between Clostridioides difficile colonization and infection
Source: Microbiome Res Rep. 2023 Dec 6;3(1):8. doi: 10.20517/mrr.2023.52 (PMC10917615; doi:10.20517/mrr.2023.52)
Supplement: Supplementary file 1 [file mrr-3-1-8-SupplementaryMaterials.pdf]

Henderickx *et al. Microbiome Res Rep* 2024;3:8
DOI: 10.20517/mrr.2023.52

Microbiome Research Reports

Supplementary Material: Fungal and bacterial gut microbiota differ between Clostridioides difficile colonization and infection

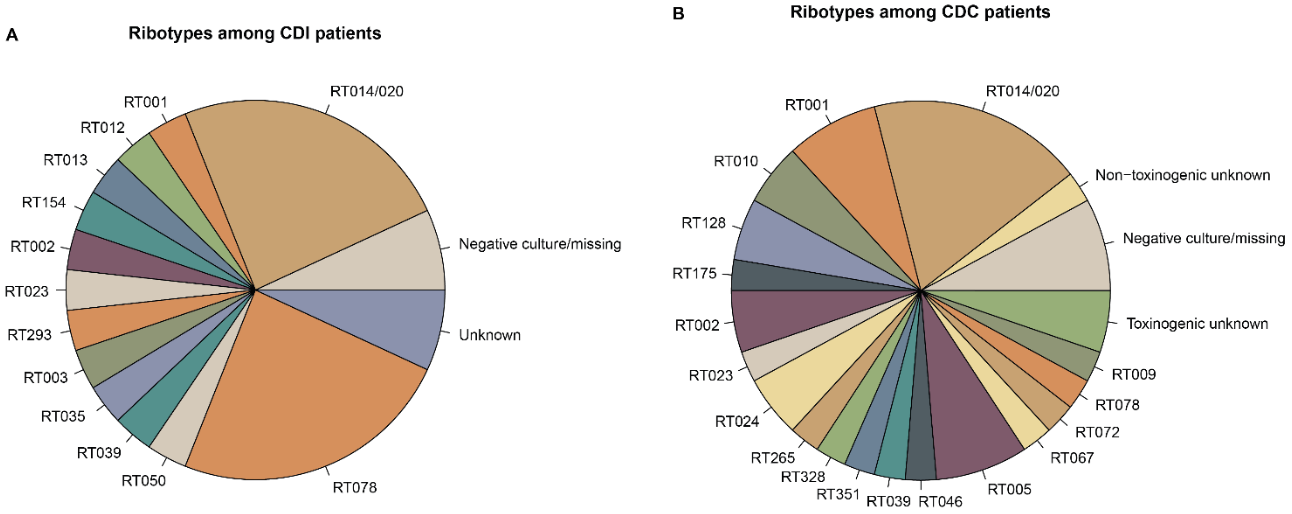

Supplementary Figure 1. Distribution of ribotypes among (A) CDI patients (n = 29) and (B) asymptomatic CDC patients (n = 38). Ribotyping was performed with capillary PCR. Ribotype 078 was significantly more prevalent in CDI (24.10%) compared to CDC (2.63%), P = 0.017 (Fisher's Exact Test) This figure is adapted with permission from Crobach *et al.* published in *Microorganisms*[29]. CDC: *C. difficile* colonization; CDI: *C. difficile* infection.

 Henderickx *et al. Microbiome Res Rep* 2024;3:8 | http://dx.doi.org/10.20517/mrr.2023.52

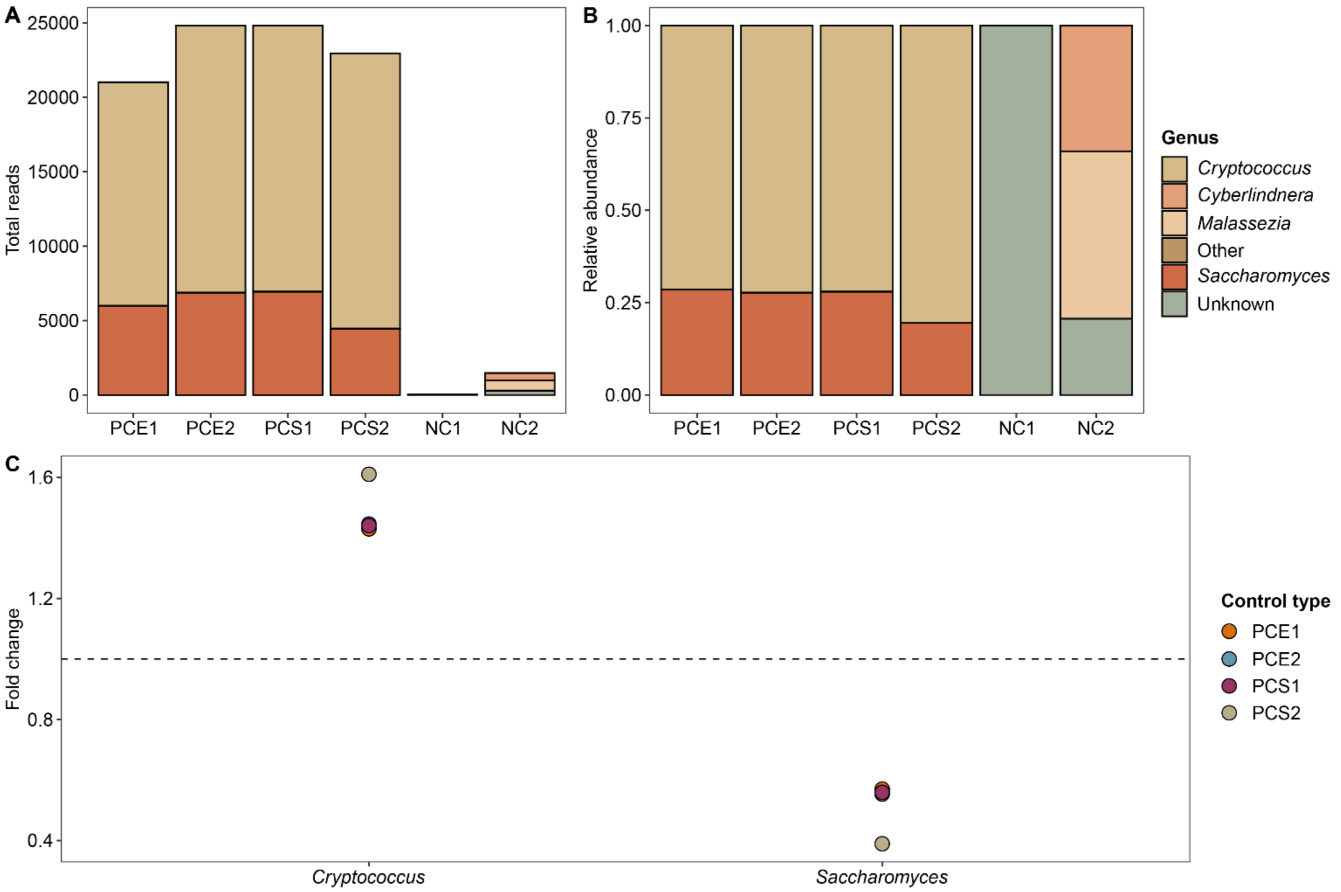

Supplementary Figure 2. Positive and negative DNA extraction controls (PCE and NC) and positive sequencing controls (PCS). Fungal taxonomic composition of the five most abundant genera shown as total reads (A) and as relative abundance (B) Taxa not belonging to the five most abundant genera were summarized into “Other”; (C) Fold change in the relative abundance of the two fungal mock genera compared to the theoretical relative abundance in the mock community (horizontal line).

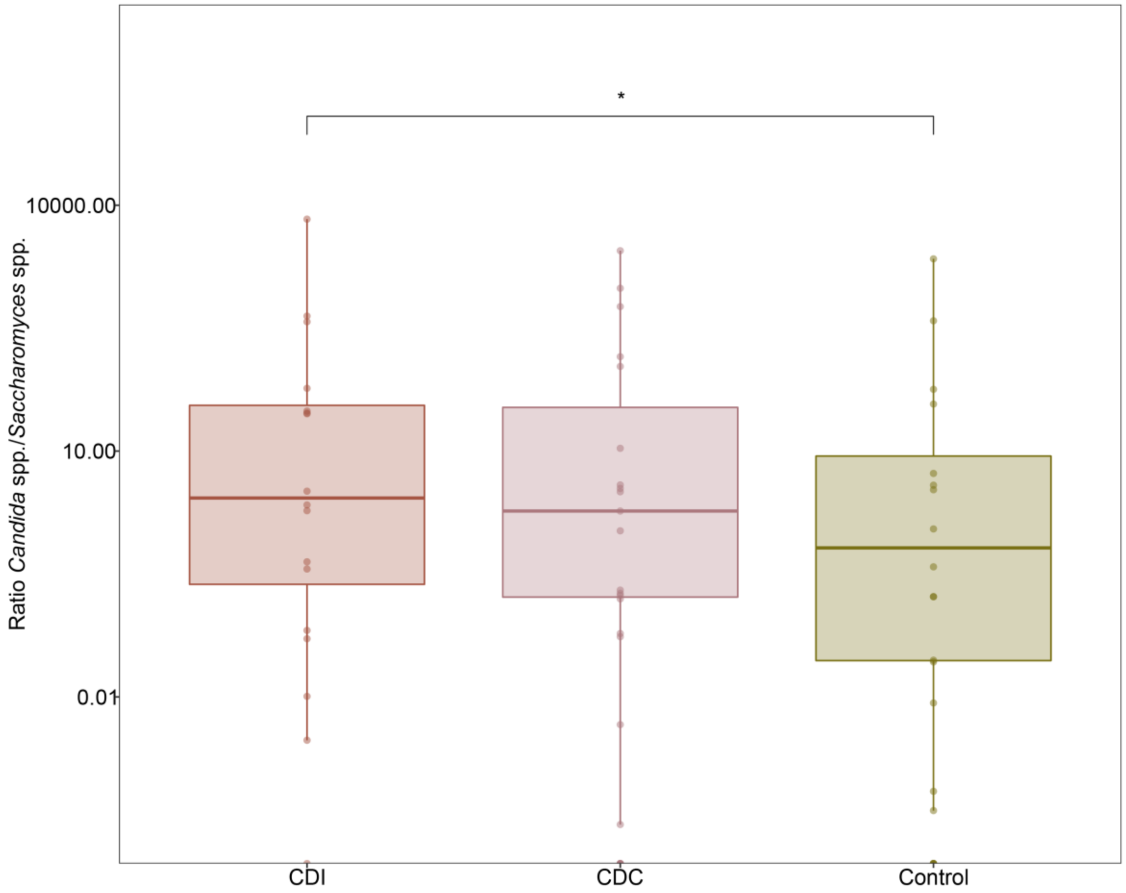

Supplementary Figure 3. Logarithmic scale of the ratio *Candida* spp. to *Saccharomyces* spp. in CDI patients, asymptomatic CDC patients and controls (Control). $^{*}P \leq 0.05$ (Wilcoxon Rank Sum Test). Non-significant results are not displayed. CDC: *C. difficile* colonization; CDI: *C. difficile* infection.

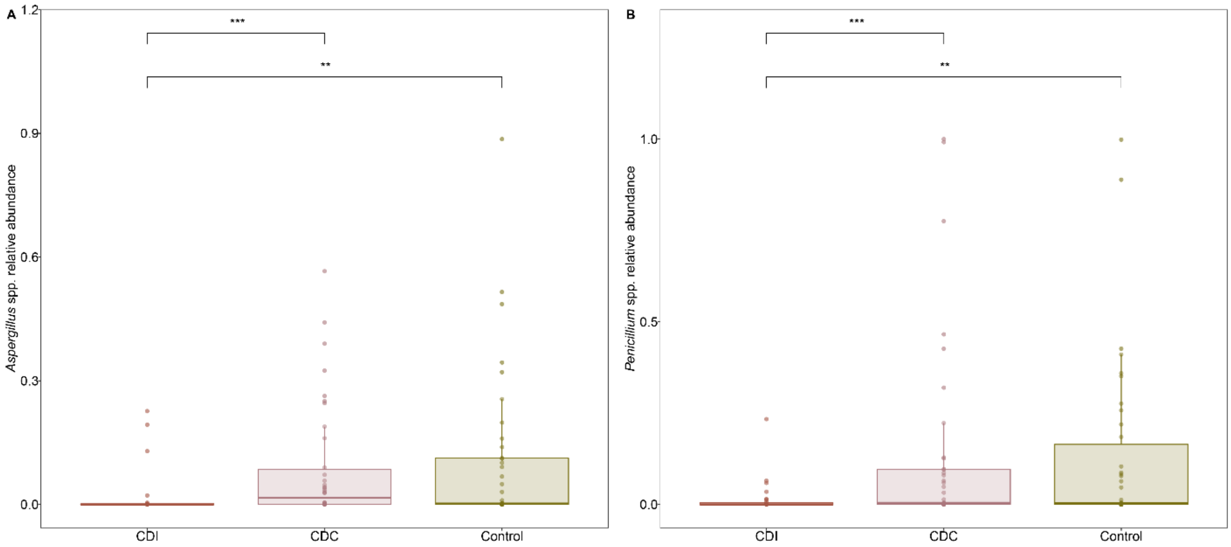

Supplementary Figure 4. Relative abundance of *Aspergillus* spp. (A) and *Penicillium* spp; (B) in CDI patients, asymptomatic CDC patients and controls (Control). Individual data points are displayed as circles. $^{***}P \leq 0.001$; $^{**}P \leq 0.01$ (Wilcoxon Rank Sum Test). CDC: *C. difficile* colonization; CDI: *C. difficile* infection.

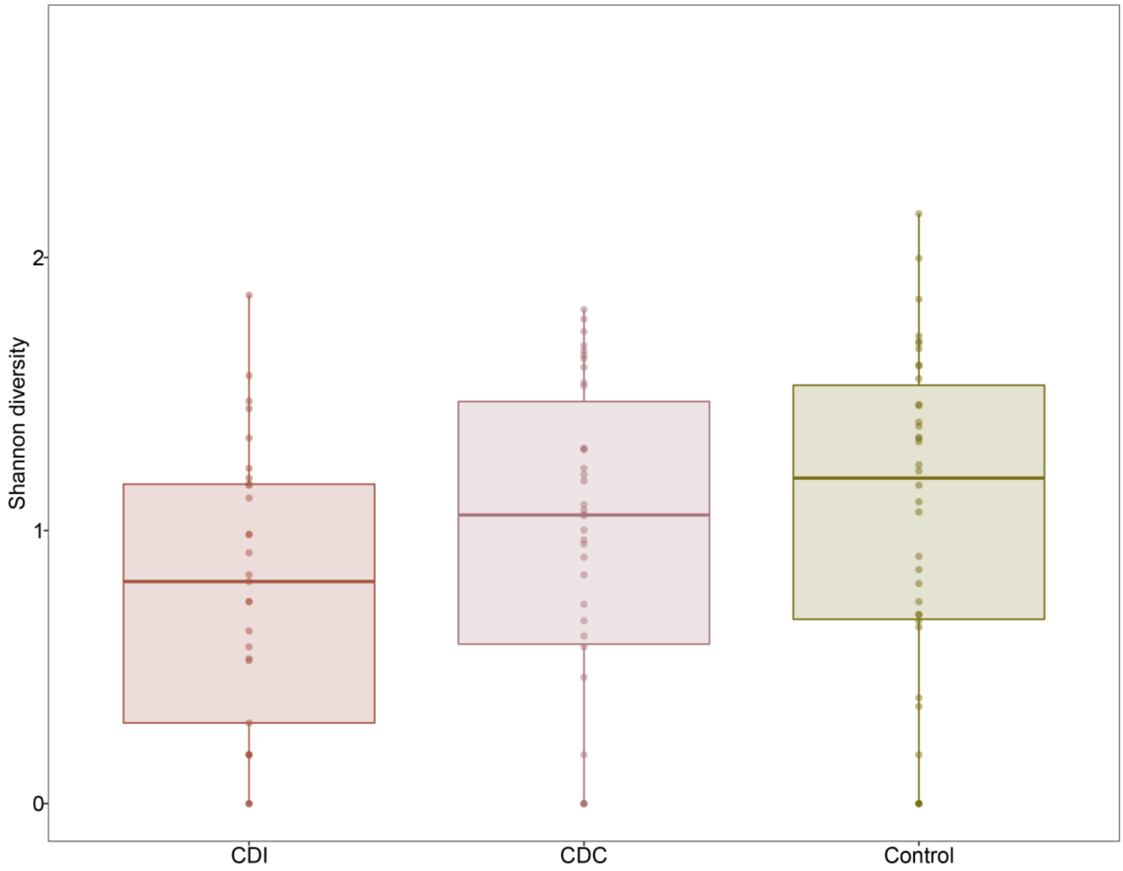

Supplementary Figure 5. Shannon diversity of the mycobiota in CDI patients, asymptomatic CDC patients and controls (Control). Non-significant results are not displayed. CDC: *C. difficile* colonization; CDI: *C. difficile* infection.

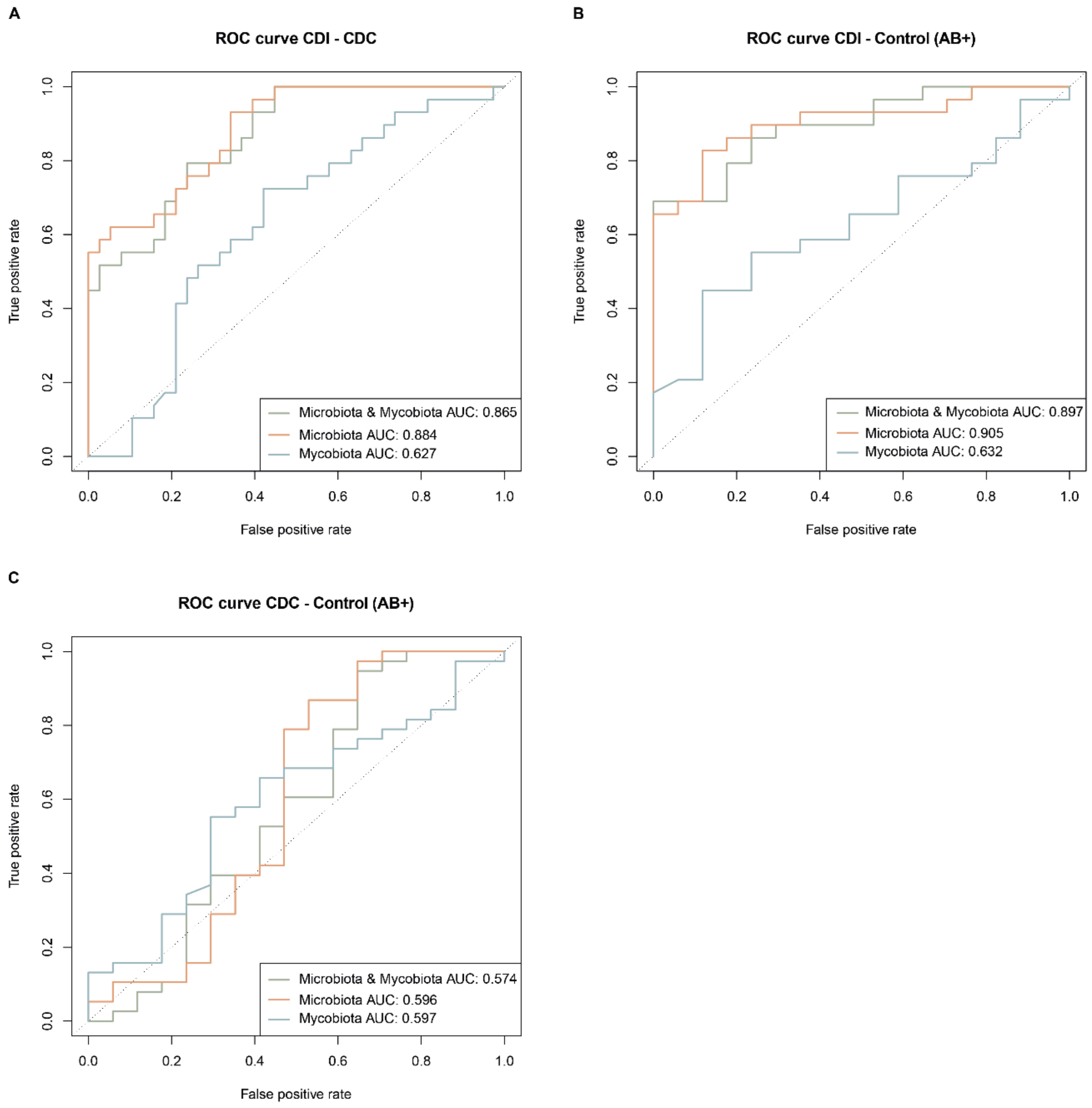

Supplementary Figure 6. Receiver Operating Characteristic (ROC) curve for the models distinguishing the groups. ROC curves are displayed for joint microbiota and mycobiota data (green), microbiota data (orange) and mycobiota data (blue) with corresponding Area Under the Curves (AUCs). (A) models distinguishing CDI patients from asymptomatic CDC patients; (B) models distinguishing CDI patients from controls who received antibiotic treatment (Control AB+); (C) models distinguishing asymptomatic CDC patients from controls who received antibiotic treatment (Control AB+). CDC: *C. difficile* colonization; CDI: *C. difficile* infection.

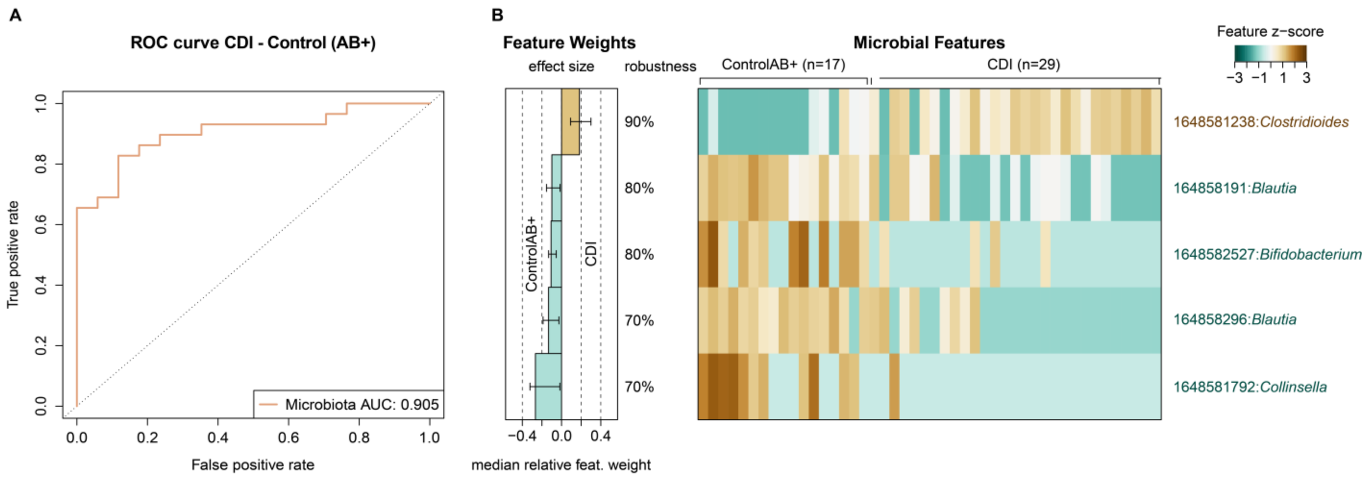

Supplementary Figure 7. Signature of gut bacterial OTUs associated to CDI patients and controls who received antibiotic treatment (ControlAB+). (A) Cross-validation accuracy of the microbiota classifier is shown in the ROC curve with the mean AUC; (B) Median relative feature weights of the selected gut bacterial OTUs show the contribution of each marker OTU to the classification. Robustness of the selected features indicates the fraction of models containing the specific feature. The normalized values of the selected features across CDI and ControlAB+ is shown in the heatmap. AUC: Area Under the Curve; CDC: *C. difficile* colonization; CDI: *C. difficile* infection; ROC: Receiver Operating Characteristic.

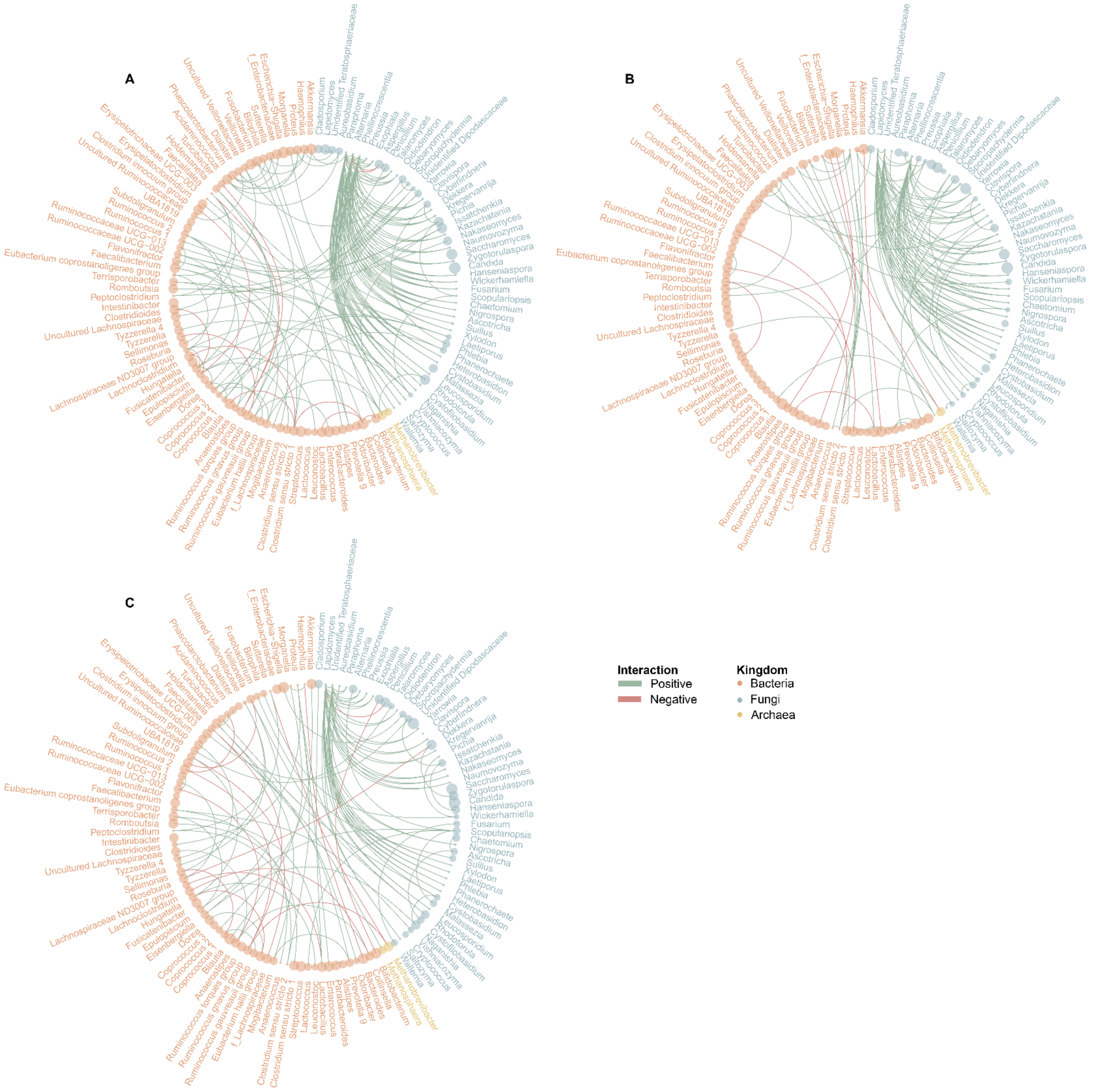

Supplementary Figure 8. Fungal-bacterial networks. Positive interactions are highlighted in green, while negative interactions are highlighted in red. Kingdoms are represented by color; mean relative abundance of the genus are represented by point size. (A) CDI patients; (B) CDC patients; (C) controls who received antibiotic treatment (ControlAB+). CDC: *C. difficile* colonization; CDI: *C. difficile* infection.

Supplementary Table 1. Subject characteristics of CDI patients, CDC patients and controls (Control)

	**CDI (*n* = 29)**	**CDC (*n* = 38)**	**Control (*n* = 38)**
**Age**	59.52 (17.640)	54.89 (19.316)	57.00 (13.981)
**Sex**			
Male	16 (55.2%)	20 (52.6%)	24 (63.2%)
Female	13 (44.8%)	18 (47.4%)	14 (36.8%)
**Hospital**			
Amphia	2 (6.9%)	24 (63.2%)	23 (60.5%)
LUMC	27 (93.1%)	14 (36.8%)	15 (39.5%)
**Previous CDI**			
Last 8 weeks	3 (10.3%)	1 (2.6%)	0 (0.0%)
>8 weeks earlier	2 (6.9%)	3 (7.9%)	0 (0.0%)
**Hospitalization (last year)**			
IBD	2 (6.9%)	7 (18.4%)	1 (2.6%)
Solid organ transplant	9 (31.0%)	7 (18.4%)	4 (10.5%)
Solid malignancy	5 (17.2%)	6 (15.8%)	11 (28.9%)
Hematological malignancy	5 (17.2%)	0 (0.0%)	2 (5.3%)
**Previous medication use (last three months)**			
Antibiotics*	28 (96.6%)	23 (60.5%)	17 (44.7%)
Immunosuppressants	19 (65.5%)	16 (42.1%)	11 (28.9%)
PPI or antacids	23 (79.3%)	27 (71.1%)	18 (47.4%)
Chemotherapy	7 (24.1%)	2 (5.3%)	4 (10.5%)

CDC: *C. difficile* colonization; CDI: *C. difficile* infection. *Previous antibiotic use uncertain in six CDC samples (15.8%) and six control samples (15.8%). This table is adapted with permission from Crobach *et al.* published in *Microorganisms*[29].
